# On the Kinematic Motion Primitives (kMPs) – Theory and Application

**DOI:** 10.3389/fnbot.2012.00010

**Published:** 2012-10-12

**Authors:** Federico L. Moro, Nikos G. Tsagarakis, Darwin G. Caldwell

**Affiliations:** ^1^Department of Advanced Robotics, Istituto Italiano di TecnologiaGenova, Italy

**Keywords:** human motion analysis, kinematic Motion Primitives (kMPs), combination of periodic and discrete movements, COMAN robot, dimensionality reduction, central pattern generators

## Abstract

Human neuromotor capabilities guarantee a wide variety of motions. A full understanding of human motion can be beneficial for rehabilitation or performance enhancement purposes, or for its reproduction on artificial systems like robots. This work aims at describing the complexity of human motion in a reduced dimensionality, by means of kinematic Motion Primitives (kMPs). A set of five invariant kMPs are identified for periodic motions, and a set of two kMPs for discrete motions. It is shown how these two sets of kMPs can be combined to synthesize more complex motion as the simultaneous execution of the periodic and the discrete motions. The results reported are an evidence of the theory of Central Pattern Generators (CPG), showing its effects on the kinematics, and are related to what presented in the literature on the Motor Primitives extracted from EMG signals. Experimental tests with the COmpliant huMANoid (COMAN) were performed to show that the kMPs extracted from human subjects can be used to transfer the features of human locomotion to the gait of a robot.

## 1 Introduction

Humans are capable of performing an impressive variety of motions including locomotion, manipulation, and coordination between simultaneous locomotion with manipulation, with the control of these motions not being trivial. This paper proposes a novel method to reduce the complexity of human motion, by describing it through a series of kinematic Motion Primitives (kMPs). The kMPs are invariant waveforms, and it will be shown that a small set of kMPs is sufficient to explain a wide variety of complex coordinated motions, both periodic (e.g., walking and running), and discrete (e.g., reaching for a target with one hand). The work demonstrates that kMPs are independent of the subject and robust to disturbances. For locomotion five kMPs are identified. They describe different gaits of walking at different velocities and running, and gaits with constrained arm motion. The work is further developed to consider reaching, and two kMPs are identified for this class of motions. The kMPs extracted from both discrete and periodic motions can be combined to produce a new set of kMPs that describes the complex motion that is the simultaneous execution of the source basic motions (e.g., reaching for a target with one hand while walking). It is interesting to notice that, from the kinematic point of view, the combined motion is neither the sequencing nor the simple superposition of the source motions.

This work is relevant to the theory of Central Pattern Generators (CPG; Brown, 1911, 1912), providing evidence of its validity from the effects noticed at the level of the kinematics, and confirming its effectiveness in describing complex motions in a lower dimensionality. Previous works in the literature have used primitives to describe human or animal motion. Among these (Tresch et al., 1999; Mussa-Ivaldi and Bizzi, 2000; D’Avella et al., 2003; Ivanenko et al., 2004, 2005; Bizzi et al., 2008; Lacquaniti et al., 2012), where Motor Primitives were extracted from EMG signals. The results presented in this paper are complementary to these works. The theory of CPG hypothesizes that a limited number of control signals is generated in the spinal cord (Dimitrijevic et al., 1998; Kiehn and Butt, 2003). These signals control the contraction of the muscles, and the work on the Motor Primitives highlights this activity; the motion produced by the muscles is the object of the analysis presented in this paper, that shows the effects of the CPG at a kinematic level, by means of kMPs.

Another related work is Santello et al. (1998), that shows how to reduce the complexity of the grasping motion of a human hand by identifying a set of synergies. Synergies are closely related to the kMPs, with the main difference being their application: every synergy is related to a basic grasping mode, and a weighted combination of the synergies describes the different grasps. Soechting and Lacquaniti (1981) performed a systematic analysis of the reaching motion, measuring wrist position, and elbow angle.

The results reported can be useful in the field of neuromotor rehabilitation, performance enhancement for athletes, or in the reproduction of human motion skills in artificial systems, i.e., robots (Ijspeert et al., 2002; Degallier et al., 2008; Ijspeert, 2008; Moro et al., 2011, 2012). In particular, Moro et al. (2011) presented the kMPs of human locomotion, and their use to generate by reconstruction a human-like gait that was tested on the COMAN robot (Tsagarakis et al., 2011). Moro et al. (2012) extended this work, using the kMPs-based trajectories and scaling them in frequency to match the first resonance frequency of the mechanism. This resulted in a significant improvement of the energy efficiency. The new contribution of the research presented in this manuscript with respect to these previous studies is that it considerably extends the previous works by considering also discrete motions (reaching with a hand) and motions that are a combination of periodic and discrete movements (reaching while walking).

## 2 Materials and Methods

In this section the experiments performed will be introduced: the subjects that participated in the experiments, the set-up used to monitor the human motions, and the method used to extract the kinematic Motion Primitives (kMPs) will be explained in detail. The statistics adopted to evaluate the similarity between kMPs, and the formula to reconstruct the joint trajectories from the kMPs will be reported as well.

### 2.1 Experimental set-up

Five healthy male subjects, aged 25–28 years old, participated in the experiments. They differ in nationality, fitness level, and physical features (height: 170–185, weight: 60–90).

Movement data were collected using a Vicon MX series T motion capture system with 6 T10 infrared cameras of 1 million pixels resolution, operating at 250 Hz. Thirty-nine passive markers were attached to the subjects and used to fit a full body model. The Plug-in Gait, provided with the Vicon software, was used to derive the kinematics (i.e., 34 joint trajectories).

**Figure 1:**
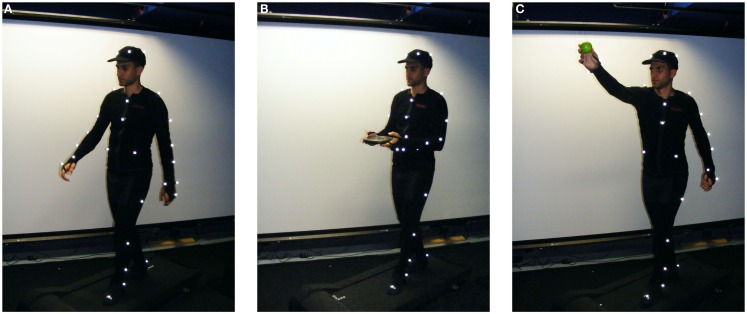
**One of the subjects (A) walking on the treadmill with no constraints, (B) walking on the treadmill holding a 5 kg load, and (C) reaching for a ball with his right hand while walking on the treadmill**.

The supplemental equipment used for the experiments includes an electrical treadmill (Christopeit Runner Pro I, velocity range: 1–12 km/h), a foam ball (6.5 cm of diameter), that was used as a target for the reaching experiments, and a standard gym 5 kg load. Figure 1 shows the set-up with one subject performing some of the gaits analyzed, wearing a black suit with the passive markers.

The set of motions recorded includes locomotion (periodic motion), reaching for a target object (discrete motion), and a combination of these two motions/tasks.

For what concerns the periodic motions, three different velocities were considered, two for walking, and one for running:

- WLS – Walking at Low Speed – 2 km/h
- WHS – Walking at High Speed – 4 km/h
- RUN – Running – 6 km/h

In addition to the unconstrained walking (hands free), the subjects were asked to walk/run while carrying an object which affected the movements of the arms. Again, three arm parameters were considered: unconstrained arms, holding an empty box, and holding a 5 kg load. These three conditions are designated as:

- Normal
- B – Holding an empty box with two hands
- 5 – holding a 5 kg load with two hands

In the notation adopted, for instance, WHSB means “walking at high speed while holding an empty box.” The combination of different speeds and constraints resulted in nine gait scenarios. For each of these gaits five trials per subject were recorded, where a trial consists of several steps taken by the subject at steady-state speed.

The above set-up allowed testing of the locomotion cycle which was considered as a period activity. Similar tests were carried out for the discrete motion of the arm. In these tests the subjects were asked to reach for a target (foam ball) with their hand. The position of the ball was fixed at the same height as the eyes of the subject, 20 cm to the left side with respect to the sagittal plane, at a reaching distance of approximately 50 cm. This made not symmetric the reaching of the ball with the left or the right hand.

- BallUp_Left – Reaching the ball position with the left hand
- BallUp_Right – Reaching the ball position with the right hand

Finally, the subjects were asked to perform a set of task motions that is a combination of the described periodic and discrete activities, i.e., reaching for the ball with either hand while walking on the treadmill (e.g., WLSBallUp_Left is “reaching for the ball with the left hand while walking at a low speed”).

From the trajectories recorded with the motion capture system for each of these scenarios, the kinematic Motion Primitives (kMPs) were extracted. In the next section the method adopted will be described.

### 2.2 kMPs extraction

In this section the methodology used to obtain the kMPs from the raw data collected will be introduced.

As anticipated, the output of the motion capture system is a set of 34 joint trajectories for each trial (7 for each arm: 3 for the shoulder, 1 for the elbow, 3 for the wrist; 7 for each leg: 3 for the hip, 1 for the knee, 3 for the ankle; 3 for the spine; 3 for the neck). Although efforts were taken to ensure that all the markers could be seen from the cameras, there were occasions when occlusions did occur. As expected this was more common in some test scenarios than others. There was some degradation of the quality of the data for fast motions (i.e., running), motions involving supporting objects that could occlude some of the markers (i.e., holding the box), or motions with self-occlusion (i.e., reaching the ball). Data sets where there was a significant loss of tracking points were neglected and not considered in the analysis. The number of valid data sets used in each experiment is reported in section 3.

Among the many dimensionality reduction techniques available in the literature, the widely used Principal Component Analysis (PCA) was chosen and applied to the sets of trajectories to extract the kMPs. PCA is a linear transformation (Pearson, 1901), and is significantly simpler than most of the other dimensionality reduction methods. Even if in certain situations PCA was proven to be not powerful enough (e.g., EMG signals), it fits well in the case of kinematic data, which are less susceptible to noise. All 34 joint trajectories describing the motion of the subjects were used as the input to PCA to maximize the information available for analysis. It was decided to apply not any normalization among the joint trajectories: this guarantees that a higher importance is given to the joint with a wider displacement.

In the case of locomotion the first five components explained about 99% of the cumulative variance. For the reaching tasks, and the combination of discrete and periodic motions (reaching while walking), two and four components were considered, respectively, for a cumulative variance explained of approximately 95%.

The components considered were called kinematic Motion Primitives (kMP), since it will be shown that they are invariant between the different subjects and for different gaits, and that they can describe in a lower dimensionality the complex motions of the subjects.

### 2.3 kMPs comparison

The analysis performed, the results of which will be shown in Section 3, aimed to investigate the effects on the extracted kMPs when the subject, the velocity, and the constraints on the arms (in the case of locomotion), or the hand used (in the case of reaching) change. The combination of periodic and discrete motions was also studied.

To compare the kMPs extracted a visual representation of the kMPs is provided for each experiment, together with related statistical information. To quantify the similarity between two sets of kMPs the maximum cross-covariance between each corresponding kMP (sliding in time) was calculated, and normalized so that the auto-covariance is 1. The delay between any two compared kMPs is also returned. This value indicates how much time-slip is needed in a signal to maximize the cross-covariance. Again this is normalized so that a slip of an entire cycle has a value of 1. An indication of the similarity (and delay) between the two entire sets is provided as the weighted average of the cross-covariance of the different kMPs. The weight used is the average of the corresponding variance explained by the kMPs compared.

Table 1 shows the statistical analysis performed, where VGj_i is the variance of the joint trajectories of Gaitj explained by the ith kMP, XGjk_i is the cross-covariance between the ith kMP extracted from Gaitj and the ith kMP extracted from theGaitk. Similarly, DGjk_i is the delay between the ith kMP extracted from the Gaitj and the ith kMP extracted from the Gaitk.

**Table 1:** **Generic table schema for the report of statistics**.

	1st	2nd	…	*i*th	Average	Sum	Weighted
Var Gait1	VG1_1	VG1_2	…	VG1_i	/	VG1_Sum	/
Var Gait2	VG2_1	VG2_2	…	VG2_i	/	VG2_Sum	/
…	…	…	…	…	/	…	/
Var Gaitj	VGj_1	VGj_2	…	VGj_i	/	VGj_Sum	/
Var average	VAv_1	VAv_2	…	VAv_i	/	VAv_Sum	/
Xcov Gait1_Gait2	XG12_1	XG12_2	…	XG12_i	XG12_Av	/	XG12_W
…	…	…	…	…	…	/	…
Xcov Gaitj_Gaitk	XGjk_1	XGjk_2	…	XGjk_i	XGjk_Av	/	XGjk_W
Xcov average	XAv_1	XAv_2	…	XAv_i	XAv_Av	/	XAv_W
Delay Gait1_Gait2	DG12_1	DG12_2	…	DG12_i	DG12_Av	/	DG12_W
…	…	…	…	…	…	/	…
Delay Gaitj_Gaitk	DGjk_1	DGjk_2	…	DGjk_i	DGjk_Av	/	DGjk_W
Delay average	DAv_1	DAv_2	…	DAv_i	DAv_Av	/	DAv_W

Other cells in the table represent the sum or the average of the corresponding row/column, apart from those in the last column. Equations (1) and (2) indicate how XGjk_W and XAv_W are defined, respectively.

(1) $$\begin{array}{c} \forall j\in \left\{1\ldots m\right\},\forall k\in \left\{j+1\ldots m\right\},\\ XGjk\text{\_}W=\left(\sum_{i=1}^{n}\;XGjk\text{\_}i\cdot \frac{VGj\text{\_}i+VGk\text{\_}i}{2}\right)\cdot \;\\ \frac{2}{VGj\text{\_}Sum+VGk\text{\_}Sum} \end{array}$$

(2) XAv_W=∑j=1m∑j=j+1mXGjk_Wm2=∑i=1nXAv_i⋅VAv_iVAv_Sum

In the same way, equations (3) and (4) define DGjk_W and DAv_W, respectively.

(3) $$\begin{array}{c} \forall j\in \left\{1\ldots m\right\},\forall k\in \left\{j+1\ldots m\right\},\\ DGjk\text{\_}W=\left(\sum_{i=1}^{n}\;|DGjk\text{\_}i|\cdot \frac{VGj\text{\_}i+VGk\text{\_}i}{2}\right)\cdot \;\\ \frac{2}{VGj\text{\_}Sum+VGk\text{\_}Sum} \end{array}$$

(4) DAv_W=∑j=1m∑j=j+1mDGjk_Wm2=∑i=1nDAv_i⋅DAv_iDAv_Sum

Notice that the statistics reported are not intended to provide a statistical proof, rather they are a quantification of what is already evident from the visual representation.

### 2.4 Trajectories reconstruction from kMPs

This section focuses on the synthesis of joint trajectories starting from the kMPs. This reconstruction is represented by:

(5) $$\begin{array}{c} \left[\;\begin{array}{c} q_{1}\\ \vdots \\ q_{i} \end{array}\right]=\left[\;\begin{array}{ccc} s_{1,1} & \cdots \; & s_{1,j}\\ \vdots & \ddots & \vdots \\ s_{i,1} & \cdots \; & s_{i,j} \end{array}\right]\times \left[\;\begin{array}{c} P_{1}\\ \vdots \\ P_{j} \end{array}\right]+\left[\;\begin{array}{c} \bar{Z_{1}}\\ \vdots \\ \bar{Z_{i}} \end{array}\right] \end{array}$$

where [*q*_1_ … *q*_*i*_] ∈ ℝ^*i*^, with *i* = 34, representing the joint trajectories vector [*P*_1_ … *P_j_*] ∈ ℝ^*j*^, with *j* equal to the number of kMPs, being the kMPs vector, and $\left[\bar{Z_{1}}\ldots \bar{Z_{i}}\right]\in {\mathbb{R}}^{i}$, with *i* = 34, being a zero offset mean vector. $\bar{Z_{i}}$ is added back to the *i*th joint trajectory (PCA was applied on the zero-mean normalized trajectories). The matrix [*s*_1,1_ … *s_i,j_*] ∈ ℝ^*i,j*^ represents the kMPs synergy map. If only a subset of the joint trajectories is required, it is possible to consider a submatrix, composed only of the rows corresponding to the joints of interest. The reference vector for the joint variables is therefore a linear combination of the kMPs through the synergetic coefficients of the matrix S. The columns of this matrix map the contribution of each primitive to the joint space. Using the extracted kMPs, the joint trajectories can be reconstructed basing on the above formula.

Some observations earned from a study on the role of the single kMPs in the overall motion will be reported in Section 4.

## 3 Results

Section 3.1 studies the effect on the kMPs extracted from periodic motions when the subjects, the velocity, and the constraints imposed change. In Section 3.2 a similar analysis is performed for the discrete motions. Section 3.3 considers the combination of periodic and discrete kMPs. The tables in these sections report only the final results of the statistics applied. An extended version of these tables with all the partial results can be found in the Appendix. Section 3.4 shows an example of joint trajectory reconstruction from kMPs. In Section 3.5 an interpretation of the kMPs is given, showing the foot trajectory when it is partially reconstructed from single kMPs or a subset of the five kMPs. Finally, in Section 3.6, the application of the kMPs to generate a valid walking for the humanoid robot COMAN is presented.

### 3.1 Experiment 1: kMPs extraction and comparison for periodic motions

**Figure 2:**
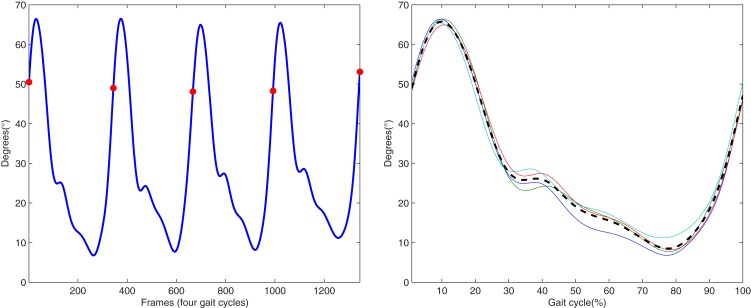
**On the left is the original left knee angle trajectory (four complete gait cycles), while on the right an averaged and normalized in time left knee trajectory is derived (dotted line) from the trajectories of the four consecutive gait cycles**.

In this subsection the kMPs extracted from different subjects, walking at different velocities or running, with different constraining conditions on the arms, are compared. In particular, in the Experiment 1.1 the kMPs of five subjects performing a low-speed walking (WLS) are compared. Next, in the Experiment 1.2, the kMPs of WLS will be compared to those of WHS. This will be extended in the Experiment 1.3 to the kMPs of running. Finally, in the Experiment 1.4 different conditions of the arms motion are considered, and the kMPs extracted from a locomotion when the arms are constrained to hold a box or a 5 kg load are compared to those of free walking. For each of the gaits in each experiment, 34 joint trajectories are recorded over a sequence of four gait cycles. These data were normalized in time (from 0 to 100% of the gait cycle) and averaged, to reduce noise (Figure 2 shows the typical results of this procedure for the left knee trajectory).

#### 3.1.1 Experiment 1.1: comparison between different subjects

**Figure 3:**
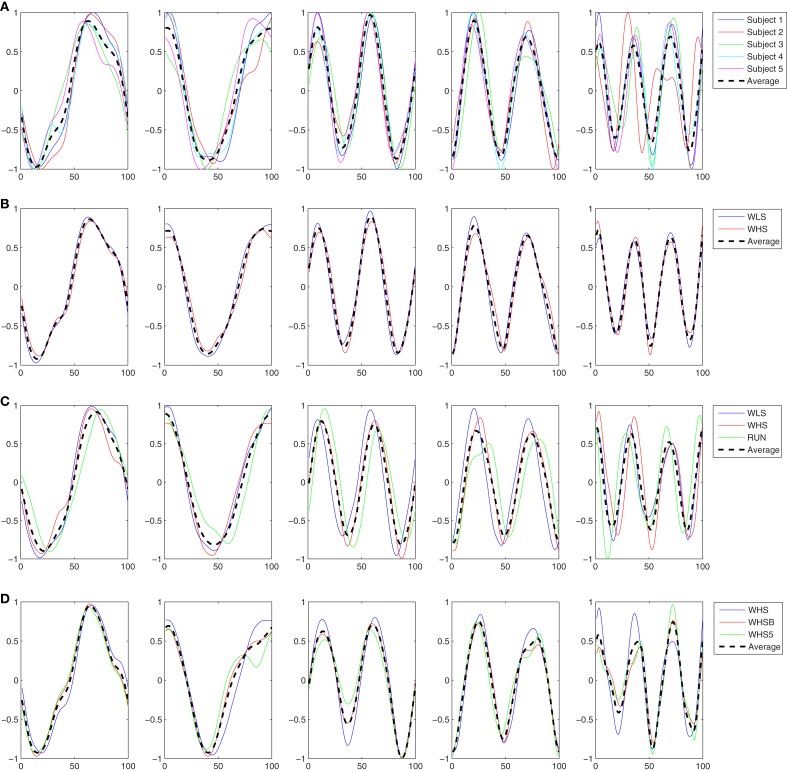
**(A)** The five kMPs extracted from the five subjects while walking at low velocity (2 km/h). **(B)** Comparison between the average kMPs of walking at low velocity (2 km/h) and walking at high velocity (4 km/h). **(C)** Comparison between the average kMPs of walking at low velocity (2 km/h), at high velocity (4 km/h), and running (6 km/h). **(D)** Comparison between the average kMPs of unconstrained walking at high velocity (4 km/h), and constrained (holding an empty box, and holding a 5 kg load) walking at high velocity (4 km/h).

In this experiment the five subjects are performing a slow walk (WLS), and all the markers were always clearly observable by the cameras. This resulted in very low noise in the joint trajectories recorded, and for this reason only one trial per subject was randomly selected and used in the analysis. Five kMPs for subject were extracted from these data, as described in Section 2.1. In Figure 3A the five kMPs are represented. The dotted line in each plot shows the average kMPs (among subjects). On the *x*-axis is the percentage of the period of the gait, from 0 to 100, while the values on the *y*-axis are between −1 and 1, indicating normalized kMPs. From these results it is clear that the first two kMPs have the same frequency as the gait, while the third and the fourth are coupled with the step (i.e., twice the gait frequency). The frequency of the fifth kMP, instead, is approximately three times the gait frequency.

Considering the general profile of the kMPs of the different subjects, it is already evident visually that the shape of the signals compared is almost identical, and the delay also is almost zero. The statistical analysis performed, the results of which are reported in Table 2 (an extended version, Table A1 in Appendix), gave a quantitative indication of the similarity between the different signals, confirming what was observed.

**Table 2:** **Walking at low velocity kMPs (5 subjects)**.

	1st	2nd	3rd	4th	5th	Average	Sum	Weighted
Var average	0.5764	0.2368	0.1346	0.0308	0.0112	/	0.9899	/
Xcov WLS	0.9648	0.9215	0.9706	0.9292	0.8506	0.9273	/	0.9527
Delay WLS	0.0320	0.0150	0.0170	0.0080	0.0300	0.0204	/	0.0249

The first five kMPs, on average, explain the 58, 24, 13, 3, and 1% of variance, respectively, for a cumulative variance explained of about the 99%. The kMPs of each subject were compared with those of all the other subjects, to obtain the cross-covariances, and the delays. The averages of these values were calculated, and it was observed that the third kMP has the least variation, with the 97% similarity. The overall similarity between the kMPs of different subjects walking at low speed was calculated as the weighted average of the values found for the single kMPs, using the average percentage of variance explained by that kMP as a weighting coefficient. This demonstrated a similarity between the kMPs of the different subjects of approximately 95%. The same method was used to evaluate the average delay, and it resulted to be about 2% of the gait cycle.

#### 3.1.2 Experiment 1.2: comparison between different walking velocities

What reported in the previous experiment was also verified for the five subjects walking at a high speed.

In this experiment the average kMPs of WLS are compared to the average kMPs of WHS (with data from five subjects). From Figure 3B it can be seen that the kMPs from gaits at different velocities are almost identical, with respect to both shape and phase. This means that the kMPs of walking, for the different subjects, are not affected by the walking speed. The results of the statistical analysis of this experiment are reported in Table 3 (an extended version, Table A2 in Appendix).

**Table 3:** **Walking at low and high velocity kMPs (5 subjects)**.

	1st	2nd	3rd	4th	5th	Average	Sum	Weighted
Var average	0.5397	0.2590	0.1486	0.0310	0.0119	/	0.9900	/
Xcov WLS_WHS	0.9955	0.9826	0.9884	0.9520	0.9551	0.9747	/	0.9894
Delay WLS_WHS	−0.0200	0	−0.0200	0	0	0.0080	/	0.0139

It can be noticed that the first kMP of WLS and WHS have a cross-covariance of more than the 99%, and that for the second and the third kMPs this value is between 98 and 99%. These three components together explain about the 95% of variance, and this results in an overall weighted average of about the 99%.

#### 3.1.3 Experiment 1.3: comparison between walking and running

The third experiment is an extension of the previous: the kMPs of running (RUN) are compared to those of walking at a low speed (WLS) and at a high speed (WHS) (Figure 3C). The data from some of the subjects were more noisy than previous tests due occlusions or slight movement of the markers caused by the stretching of the elastic suit worn by the subjects. To avoid having results corrupted by noise, the data from only three of the subjects, those whose data had the best quality, were used. The kMPs of the subjects performing the three gaits were averaged, and the resulting kMPs of WLS, WHS, and RUN were compared.

A first observation is that the similarity between WLS and WHS, that in the previous experiment with five subjects was reported to be 99%, is now about 98% (Table 4; an extended version, Table A3 in Appendix).The 1% difference is believed to be caused by the reduction in the number of subjects itself. The noise in the data of every subject, and in the resulting kMPs, is reduced in the average kMPs as the number of subjects increases.

**Table 4:** **Walking at low and high velocity and running kMPs (3 subjects)**.

	1st	2nd	3rd	4th	5th	Average	Sum	Weighted
Var average	0.5294	0.2697	0.1535	0.0279	0.0104	/	0.9908	/
Xcov WLS_WHS_RUN	0.9872	0.9305	0.9673	0.8545	0.8419	0.9163	/	0.9635
Delay WLS_WHS_RUN	0.0433	0.0066	0.0533	0.0400	0.0367	0.0360	/	0.0347

The comparison between the RUN kMPs and the WLS and WHs kMPs, considered one per time, is instead of the 95 and 96%, respectively. The average between these two values, and the one between WLS and WHS, represents the similarity between the different locomotion gaits, and is more than 96%.

#### 3.1.4 Experiment 1.4: comparison between unconstrained and constrained walking

The final experiment into periodic motions considered a comparison between unconstrained walking, as explored in each of the previous experiments, and walking while holding an object with the two hands, which introduced a constraint on the ability to swing the arms (Figure 3D). Two cases were analyzed. In the first the subjects were asked to hold an empty box, which introduced a constraint on the motion, but negligible physical loading. In the second scenario the object was a 5 kg load, which not only constrained the motion of the arms, but also introduced a constant force due to gravity pushing the hands down. The goal of this test was to determine if the kinematics of the motion, analyzed by means of kMPs, was affected by constraint imposed with this holding of objects.

From the results reported in Table 5 (an extended version, Table A4 in Appendix) it can be seen that the similarity between the gaits is high, and this means that the respective kMPs are not much affected by the constraints imposed. The first kMP in the three cases is almost identical: in the three comparisons the cross-covariance is always of about 99%. The average cross-correlation of the other kMPs slightly reduces, but it remains between the 90% of the fifth kMP and the 96% of the fourth kMP.

**Table 5:** **Walking with no constraint, holding an empty box and holding a 5 kg load kMPs (3 subjects)**.

	1st	2nd	3rd	4th	5th	Average	Sum	Weighted
Var average	0.5129	0.2697	0.1633	0.0336	0.0097	/	0.9893	/
Xcov WHS_WHSB_WHS5	0.9925	0.9369	0.9545	0.9590	0.9022	0.9490	/	0.9566
Delay WHS_WHSB_WHS5	0.0200	0	0.0067	0	0.0067	0.0067	/	0.0116

The resulting overall similarity among the different gaits is more than 96%, indicating that the kMPs of locomotion remain invariant even if a constraint on the swing motion of the arms is imposed.

### 3.2 Experiment 2: kMPs extraction and comparison for discrete motions

The second set of experiments was focused on the discrete motion of reaching for a target with the hand. The target was a foam ball with a diameter of 6.5 cm, suspended in front of the subject at the same height as the eyes of the subject. The ball was 20 cm to the left of the centerline, at a distance from the subject of about 50 cm. The subjects were asked to assume an initial pose (standing, arms by their sides), to reach for the target with one hand, and finally to move back to the initial position. The data collected in these experiments were affected by self-occlusion: when the subjects were asked to reach for the target with the hand, some of the markers placed on their trunk were occluded to the cameras by their own arm. For this reason the data coming from only two subjects, which were considered clean enough, were used in the analysis.

Both reaching with the left hand and reaching with the right hand were recorded (since the ball was not located in the sagittal plane of the subjects, their motions of reaching with the left and the right hand were not symmetric).

In the Experiment 2.1 the kMPs of the different subjects performing the reaching motion with the left hand will be compared. Next, similarly to what done for the periodic motions, in the Experiment 2.2 the kMPs of reaching with the left hand will be compared to those of reaching with the right hand.

#### 3.2.1 Experiment 2.1: comparison between different subjects

In this experiment the subjects were asked to stand in a neutral initial position in front of the target as previously described, to reach for it with the left hand, and to move back to the initial position. No constraint on the timing to complete the motion was imposed.

**Figure 4:**
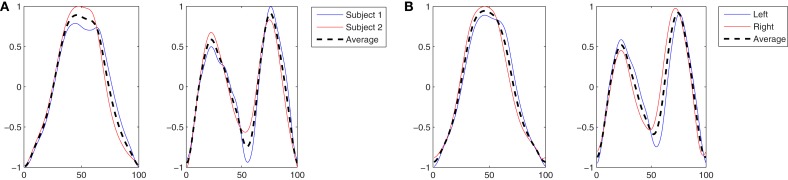
**(A)** The two kMPs extracted from the two subjects reaching a target with the left hand. **(B)** Comparison between the average kMPs of reaching a target with the left and the right hand.

In Figure 4A the two kMPs, that together explain on average the 97% of variance, are shown. The first of these kMP has a single peak, while in the second kMP two peaks are present: each peak takes approximately 50% of the total reaching time, and the first peak has an amplitude that is about 25% smaller than the amplitude of the second peak.

As reported in Table 6 (an extended version, Table A5 in Appendix), the weighted average cross-covariance between the kMPs is about 96%, showing that the similarity between the reaching motion of the two subjects, in terms of kMPs, is significant. It is also interesting to notice that in all cases there is no delay between the kMPs of the different subjects. This indicates that what stated on the periodic motions (described by five invariant kMPs) can also be extended to the discrete motions (described by two invariant kMPs).

**Table 6:** **Reaching a target with the left hand (2 subjects)**.

	1st	2nd	Average	Sum	Weighted
Var average	0.8201	0.1510	/	0.9711	/
Xcov BallUp_Left	0.9662	0.9253	0.9457	/	0.9598
Delay BallUp_Left	0	0	0	/	0

#### 3.2.2 Experiment 2.2: comparison between reaching with the left and the right hand

Having demonstrated that the kMPs of different subjects performing the same discrete motion are closely related, two reaching motions that are slightly different were compared. The first set of kMPs came from the previous experiments, and describes the reaching motion with the left hand. The second is the set of kMPs that describe reaching with the right hand, and was extracted from the data of the same subjects. The kMPs extracted are reported in Figure 4B.

Table 7 (an extended version, Table A6 in Appendix) shows that the first kMP has a cross-covariance of about the 97%, while in the second instance this value is about 87%. This results in a weighted average of almost 96%, with no noticeable delays between the kMPs. It is now reasonable to state that also the discrete motions can be described by means of a set of two invariant kMPs. It is interesting to note that, although reaching with the left and right hands are not symmetrical (the location of the target ball was not in the sagittal plane of the subjects), this difference is not reflected in the extracted kMPs, which are once again the same.

**Table 7:** **Reaching a target with the left and the right hand (2 subjects)**.

	1st	2nd	Average	Sum	Weighted
Var average	0.8326	0.1332	/	0.9658	/
Xcov BallUp	0.9693	0.8745	0.9219	/	0.9562
Delay BallUp	0	0.0100	0.0050	/	0.0014

### 3.3 Experiment 3: Combination of periodic and discrete kMPs

After having shown that both periodic and discrete motions can be described by a small set of invariant kMPs, in this last set of experiments the focus is on more complex motions that combine a periodic and a discrete basic task. The subjects were asked to perform the reaching motion described above (Experiment 2), while walking on the treadmill at a low speed (Experiment 1). The two subtasks were synchronous: the subjects were asked to complete the reaching task (both the motion to reach for the ball, and the motion back from the ball to the initial position) in a time that was approximately the same as the period of two steps, that is a full gait cycle. The subjects reported that this requirement was easy to satisfy: a motivation for this observation can be found in Michaels and Bongers (1994), Sternad et al. (2000), and De Rugy and Sternad (2003).

It is important to notice that the combination of tasks analyzed in this experiment is different from the analysis available in the literature: it is not a sequencing of tasks, and not even a simple superposition, since the swinging motion (coming from the walking) of the arm used for reaching is suppressed in the period of time when the subject is actually performing the reaching task.

#### 3.3.1 Experiment 3.1: comparison between different subjects

**Figure 5:**
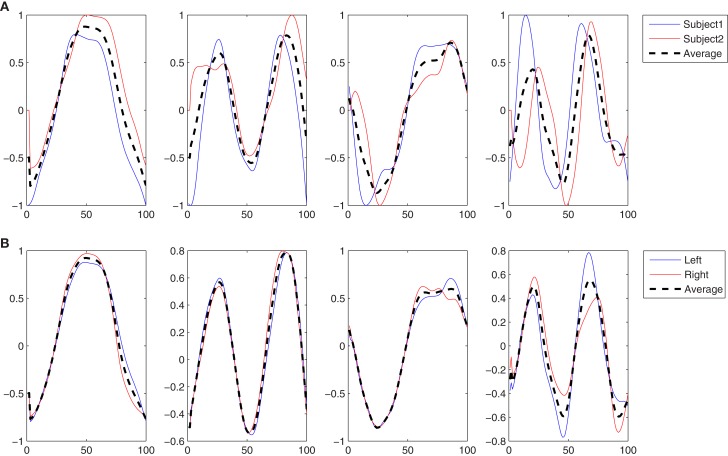
**(A)** The four kMPs extracted from the two subjects reaching a target with the left hand while walking. **(B)** Comparison between the average kMPs of reaching a target with the left and the right hand.

In the same way as it was in the Experiment 2.1 the subjects were asked to reach for the target ball with the left hand. Differently from the previous case, for these experiments the reaching task had to be accomplished while walking at low speed on the treadmill. The complex reaching while walking task was then a combination of the periodic motion WLS presented in the first set of experiments, and the discrete motion BallUp_Left from the second set of experiments. The kMPs extracted from the joint trajectories of the two subjects performing this composed task are reported in Figure 5A.

The first four kMPs together explain on average the 97% of variance. The overall weighted average cross-covariance is 90%, as reported in Table 8 (an extended version, Table A7 in Appendix), and this proves that the motion of the two subjects, analyzed by means of the kMPs, has a good level of similarity.

**Table 8:** **Reaching a target with the left hand while walking (2 subjects)**.

	1st	2nd	3rd	4th	Average	Sum	Weighted
Var average	0.7770	0.0946	0.0720	0.0277	/	0.9689	/
Xcov WLSBallUp_Left	0.9347	0.6070	0.8971	0.8697	0.8271	/	0.9004
Delay WLSBallUp_Left	−0.0200	−0.0500	−0.0700	−0.0100	0.0375	/	0.0264

#### 3.3.2 Experiment 3.2: comparison between reaching with the left and the right hand while walking

In this experiment the average kMPs of reaching for the target with the left hand while walking on the treadmill at a low speed (WLSBallUp_Left) are compared to the corresponding kMPs of reaching for the target with the right hand while walking on the treadmill at a low speed (WLSBallUp_Right), as reported in Figure 5B.

The first three kMPs are almost identical, with a cross-covariance of 98, 98, and 99%, respectively (Table 9; an extended version, Table A8 in Appendix). The overall weighted average is thus approximately 98%, while the delay approaches 0%. Also in this case, the motion for reaching with the left hand, and the motion for reaching with the right hand were not symmetrical, but this difference does not reflect on the corresponding kMPs extracted, that once again resulted to be the same.

**Table 9:** **Reaching a target with the left and the right hand while walking (2 subjects)**.

	1st	2nd	3rd	4th	Average	Sum	Weighted
Var average	0.7837	0.0895	0.0677	0.0276	/	0.9672	/
Xcov WLSBallUp	0.9827	0.9759	0.9862	0.8654	0.9526	/	0.9803
Delay WLSBallUp	0	0	0	−0.0200	0.0050	/	0.0006

#### 3.3.3 Experiment 3.3: comparison between reaching while walking and reaching and walking separately

**Figure 6:**
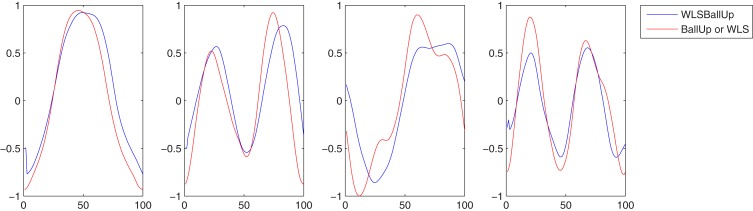
**Comparison between the average kMPs of reaching a target with the hand while walking (blue line) and the average kMPs of reaching (red line, first and second graph), and walking (red line, third and fourth graph)**.

This experiment was the most challenging among those presented in this paper. What was compared, in fact, was not a set of kMPs from the same motion, but the kMPs of reaching while walking, with the kMPs of reaching and the kMPs of walking, separately. A correspondence between the kMPs extracted from the reaching while walking motion, and some of the kMPs extracted from the two simpler motions was noticed. More specifically, the first and the second kMPs of WLSBallUp (blue line in the first and second graph, Figure 6) were very similar to the first and the second kMPs of BallUp (red line in the first and second graph, Figure 6), respectively. The third and the fourth kMPs of WLSBallUp (blue line in the third and fourth graph, Figure 6), instead, were very similar to the first and the fourth kMPs of WLS (red line in the third and fourth graph, Figure 6), respectively.

From Table 10 it can be noticed that the cross-correlation between the kMPs is high: the maximum value observed is the one corresponding to the comparison between the first kMP of WLSBallUp and the first kMP of BallUp, which is 98%, while the minimum, which is for the comparison between the second kMP of WLSBallUp and the second kMP of BallUp, is 81%. The other two comparisons resulted in a cross-correlation of 95 and 93%, respectively, for an overall weighted average of about 96%. The weights used for the definition of this synthetic indicator of similarity, in this case, are the amount of variance explained by the single kMPs of the WLSBallUp only, since the second set of kMPs is a combination of the sets of kMPs of two different motions. It can also be observed that the weighted average delay between the two sets of kMPs under comparison is less than the 1% of the cycle period.

**Table 10:** **Combination of the kMPs of walking and the kMPs of reaching (2 subjects)**.

	1st	2nd	3rd	4th	Average	Sum	Weighted
WLS	/	/	1st kMP	4rd kMP	/	/	/
BallUp	1st kMP	2nd kMP	/	/	/	/	/
Var WLSBallUp	0.7837	0.0895	0.0677	0.0276	/	0.9672	/
Xcov Combination	0.9762	0.8085	0.9480	0.9339	0.9167	/	0.9588
Delay Combination	0	0.0300	0.0700	0	0.0250	/	0.0077

The results achieved in this experiment show how the kMPs extracted from the periodic and the discrete motions can be combined to produce more complex motions that are a combination of the simpler source motions. The resultant complex motions guarantee the execution of the two tasks, one periodic and the other discrete, and can be described neither by the simple superposition, nor by the sequencing of the motions to perform the two tasks separately. The kMPs of this complex motion, instead, are a proper combination of the kMPs extracted from the two basic tasks.

These results allow certain conclusions to be drawn on how human motion control and coordination is performed: according to this study five kMPs can effectively describe the different periodic motions, while two kMPs can be responsible for synthesizing different discrete motions. A combination of kMPs of the periodic and discrete motions may be used to generate different complex motions, that simultaneously accomplish the periodic and discrete tasks.

### 3.4 Example of joint trajectory reconstruction from kMPs

In this Section the contribution of the kMPs to the joint trajectories is analyzed, showing the left knee trajectory generated by reconstruction from single kMPs and from subsets of the five kMPs.

**Figure 7:**
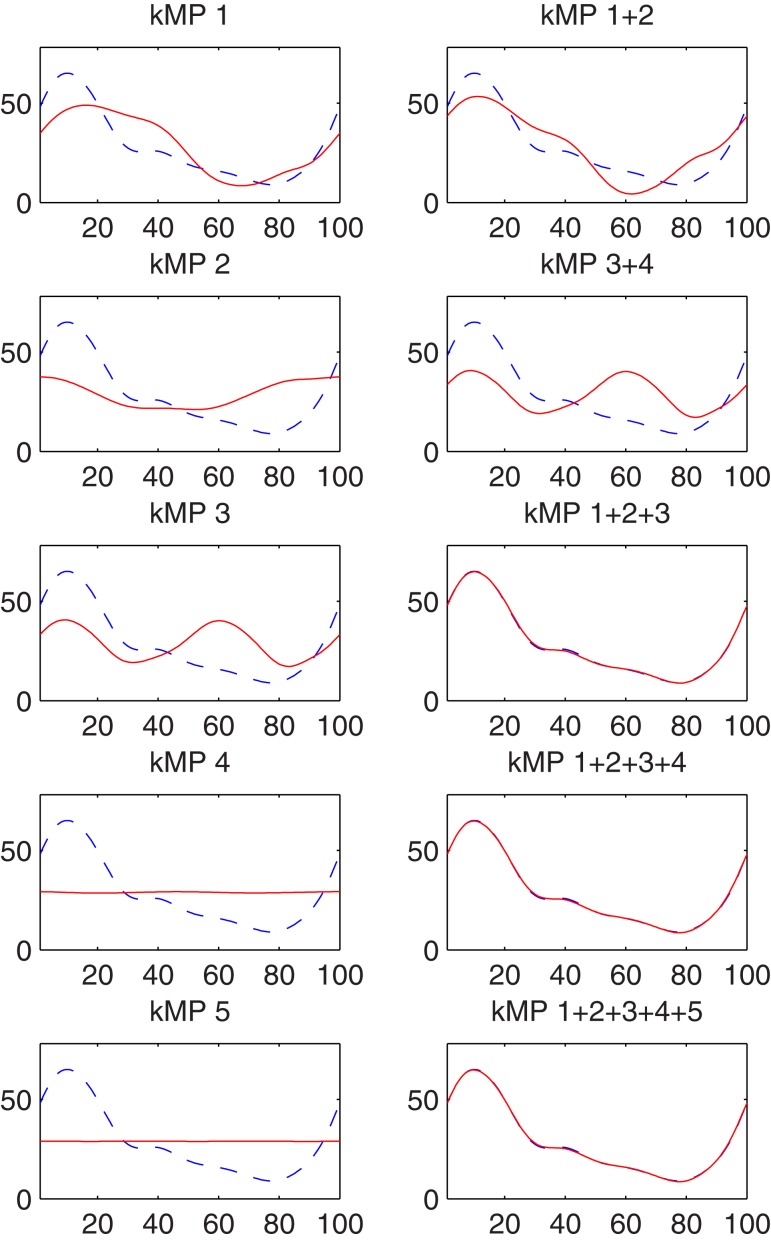
**Left knee trajectory generated by reconstruction from kMPs (solid line) compared to the original left knee trajectory (dotted line)**. On the x-axis the percentage of a gait cycle (from 0 to 100%), and on the y-axis the knee angle in degrees.

Figure 7 shows that no single kMP is sufficient to guarantee a good accuracy in the reconstruction. In this specific example, the first three kMPs describe most of the original motion, and when they are considered together the trajectory reconstructed is very close to the original one, while the introduction of the fourth and the fifth kMPs only brings an improvement that is negligible.

### 3.5 Partial reconstruction of the foot trajectory from kMPs

**Figure 8:**
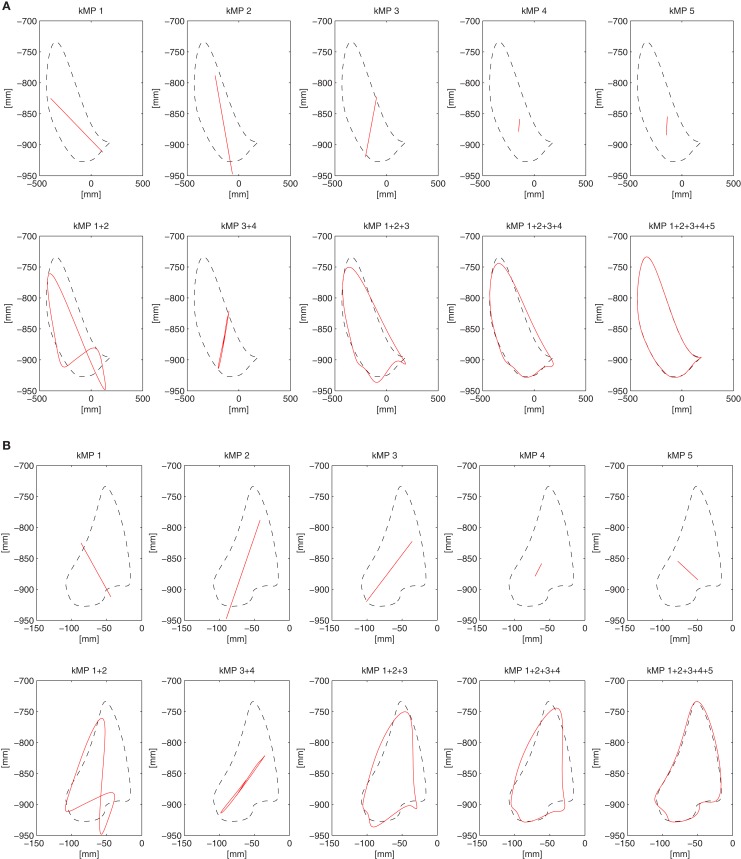
**Partial reconstruction from kMPs of the right foot trajectory (A) projected on the sagittal plane, and (B) projected on the coronal plane**.

This section considers the contribution of the single kMPs to the foot trajectory. In Figure 8 the blue line is the right foot trajectory (relative to the pelvis) of a subject walking at low speed, projected on the sagittal plane (Figure 8A) and on the coronal plane (Figure 8B), respectively. In both figures the first row of graphs compare the foot trajectory reconstructed from an individual kMP (labeled on top), in red, with respect to the original trajectory. The second row compares foot trajectories reconstructed from a combinations of kMPs, in red, again with respect to the original trajectory. The second row, instead, compare the foot trajectory reconstructed from a set of kMPs (label on top), in red, to the original trajectory.

The displacement of the foot trajectory on the longitudinal axis is about 20 cm, while on the medial axis it is about 60 cm, and on the transverse axis it is about 10 cm. The first two kMPs together are enough to give a hint on what the range of motion of the foot is. By including the third kMP the trajectory reconstructed has already a similar shape to the one of the original trajectory.

It can be also noticed that, if all the five kMPs are used, the resulting foot trajectory obtained by reconstruction is almost identical to the original trajectory.

### 3.6 Application: Reconstruction from kMPs of a human-like walking for the COMAN robot

**Figure 9:**
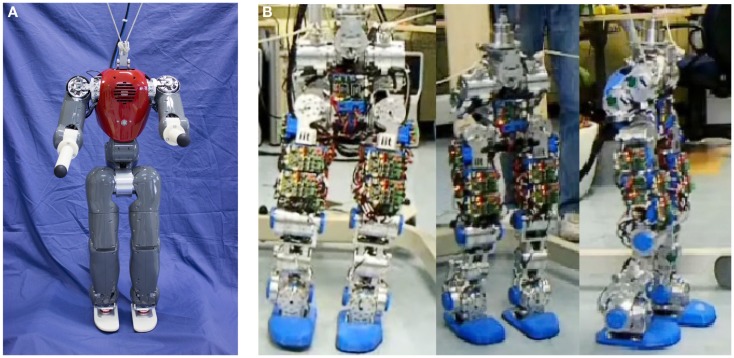
**(A)** The COMAN robot as it currently appears. **(B)** Snapshots from the video of the COMAN robot walking human-like, with CoM trajectory reconstruction from kMPs. The height of the COMAN lower body, from the foot to the waist, is 671 mm, with a maximum width and depth (at the hips) of 176 and 110 mm, respectively. The total lower body weight is 17.3 kg, with each leg weighing approximately 5.9 kg, and the waist section, including the hip flexion motors, weighing 5.5 kg. The leg of COMAN incorporates two series elastic (SEA) actuation units, which are placed at the knee flexion and the ankle dorsiflexion joints.

This section reports the use of kMPs to generate a walking for the COmpliant huMANoid (COMAN) robot (Tsagarakis et al., 2011), under development in the Department of Advanced Robotics at the Istituto Italiano di Tecnologia (IIT). Figure 9A shows COMAN as it currently appears. At the moment when the experiments presented in this paper were performed, a first prototype of the robot, composed by the lower body only, was available. Only the trajectories for the joints of the legs hence were generated. The application to generate trajectories for a humanoid robot from kMPs, though, is not the main focus of this paper: more details on the COMAN robot walking human-like can be found in Moro et al. (2011, 2012).

When the kMPs are extracted from the joint trajectories of a subject, the coefficients of the matrix S in equation (5) can be used to reconstruct the trajectories for the same specific subject. It is not easy then to use these trajectories as they are, and fit them on a robot that has a kinematics which is slightly different. Moreover, there are some constraints, in particular on the range of motion of some of the joints of the robot, that made it necessary to look for an alternative solution. It was decided, hence, to consider the center of mass (CoM) trajectory, which was provided by the Vicon motion capture software according to an estimation of the human mass distribution. Since the CoM trajectory and the joint trajectories are coupled, if the CoM trajectory is added to the set of joint trajectories, the kMPs extracted remain the same. In this way the coefficients to reconstruct the CoM itself are available. The CoM reconstructed was scaled down, and, via inverse kinematics, a set of joint trajectories that satisfies it was derived. The gait generated by these trajectories was tested on the COMAN robot. Figure 9B shows a series of snapshots of COMAN walking using kMPs.

Among the others, three are the main features that characterize human walking if compared to the typical robot locomotion: the heel-strike, the toe-off, and the straight knees. Mechanical constraints (rigid foot) represent a limitation for the COMAN robot for what concerns the first two features. The third feature (straight knees), instead, was observed in the gait generated by reconstruction from kMPs, though not imposed in any way.

**Figure 10:**
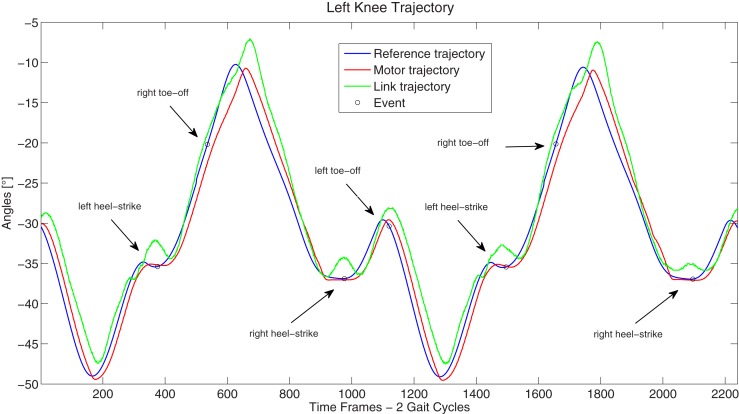
**Left knee reference, motor, and link trajectories**.

The resultant gait, hence, was observed to have strong human-like traits (this can be noticed in the video of the COMAN robot walking human-like): the variation of the CoM height was relatively large compared to the almost flat CoM motion of most humanoids, and the knee straighten to −5°, which is a strong contrast to the very bent knee walk typically observed in robot locomotion, where the knee angles are never greater than −25° throughout the gait cycle (in Figure 10 the left knee trajectory is reported).

**Figure 11:**
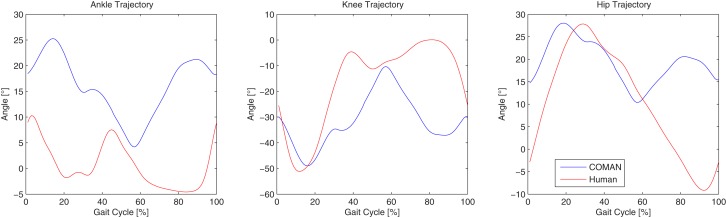
**Comparison between left ankle, knee, and hip trajectories of a human subject, and those of the COMAN robot walking human-like**.

Figure 11 shows the left ankle, knee, and hip trajectories of a human (red line), and those of COMAN walking in a human-like manner (blue line). In the second graph it can be seen that COMAN’s knee trajectory show the described straightening from approximately −50° to about −5°, following a profile that is similar to that of the human. After this knee straightening the robot knee has an increasing bend angle that is not present in the human. This motion is due to the rigid feet of the robot (no toe flexing is possible), that presents a non-negligible limitation to the motion of the robot. Indeed this lack of flexibility in the foot limits the step length of the robot, and made it necessary to always keep the orientation of the foot parallel to the ground. These constraints also reflect on the hip and ankle trajectories: the range of motion of the hip joint is limited to only 15°, and the ankle trajectory is roughly the same as the opposite of the knee trajectory.

## 4 Discussion

Humans can perform diverse intricate tasks involving a vast range of different motions, many of which are not preplanned. This flexibility, and the variety of possible solutions, means that control paradigms are complex. Previous works, mostly focused on hand grasping motions (Santello et al., 1998), or based on the monitoring and analysis of EMG signals (D’Avella et al., 2003; Ivanenko et al., 2004, 2005) have shown that this problem can be tackled using dimensionality reduction techniques. The work presented in this paper, in a similar manner, investigates the existence of kinematic Motion Primitives (kMPs) in the human motion control.

In Section 2 details on the experiments were introduced, including specification of all the materials used, and the procedures adopted to perform the analysis. From these data, five kMPs were extracted for locomotion, and a further two kMPs were presented for reaching motions. Comparative studies were carried out among different subjects. From this analysis it was demonstrated that, although joint trajectories appear different for each subject, they can actually be described by the same small set of invariant kMPs. It has also been shown that velocity (slow walking, fast walking, and running) does not affect the kMPs, which remain unchanged even after the introduction of constraints to the motion of the arms.

For discrete (non-cyclic) motions, it has been proven that reaching with either the left or the right hand (even when the target is not in the sagittal plane of the subject) results in the same set of kMPs. These results were reported in Section 3, along with pertinent detailed statistical data.

The final experiment sought to show how the kMPs extracted from periodic and discrete motions can be combined to produce more complex movements (e.g., reaching for a target with either hand while walking). It was discovered that the intricate motions analyzed are neither the sequencing of basic motions, nor their simple superposition. What was observed is that the kMPs of the complex motion are a subset of the kMPs extracted from the periodic and the discrete motions performed separately. This suggests that humans can produce different complex motions through the synergetic combination of a very small set of kMPs. These results, in some degree, confirm ideas hypothesized by the original CPG theory (Brown, 1911, 1912), and move some way toward explaining how humans can handle the complexity of motions experienced in daily tasks. For this reason it may be appropriate that this work be classified together with those on CPG, that focus on the signals generated in the spinal cord (Dimitrijevic et al., 1998; Kiehn and Butt, 2003), and those on Motor Primitives (Tresch et al., 1999; Mussa-Ivaldi and Bizzi, 2000; D’Avella et al., 2003; Ivanenko et al., 2004, 2005; Bizzi et al., 2008), that observe the effects of the signals from the CPG at the level of muscle activation. This work is a further step that observes the effects of the muscle activation, driven by CPG signals, at a kinematic level, for both periodic and discrete motions. Although originally the CPG theory was used to describe periodic movements only (Delcomyn, 1980; Grillner, 1985, 2006; Marder and Bucher, 2001; Ronsse et al., 2009) extended it to the discrete movements also. In the work of Schaal et al. (2004), Van Mourik and Beek (2004), Hogan and Sternad (2007), Sternad (2008), Dégallier Rochat and Ijspeert (2010), and Dégallier Rochat et al. (2011) it is possible to find important contributions to the on-going discussions around the possibility of describing both periodic and discrete movements by means of a unified theory.

The differences between the kMPs compared in this study are always relatively small (never below 90% of similarity). This is confirmed by the results of the statistical analyses, which suggest that this variation mostly arises from the noise in the data collection of the joint trajectories. Evidence to support this is the improvement achieved when the number of subjects increases. The presence of this noise was not unexpected, and comes primarily from a less than perfectly accurate tracking system, and from unavoidable small movements of the markers on the body of the test subjects, due to the elasticity of the skin and the suit. Not withstanding these problems, the accuracy reached in the extraction of the kMPs is significantly higher than those reported in the literature for Motor Primitives extracted from EMG signals. In the latter case, in fact, the signal to noise ratio is lower.

In spite of the effort made to investigate the nature of the coefficients of matrix *S* [equation (5)], it was hard to identify a regular pattern in the synergetic mapping of the kMPs to the joint trajectories. This is reasonably because there are significant differences in the joint trajectories among subjects/gaits. As it has been shown, the kMPs extracted are always very similar. This means that what produce the diverse gaits are the coefficients of matrix *S*. Some interesting experimental results were collected to investigate the contribution of the single kMPs to the motion of the lower limbs for walking. A simple model of the legs was developed, and the hip, knee, and ankle trajectories were reconstructed from the single kMPs, and tested on the simulation model. This analysis revealed that the first two kMPs are mainly responsible of the alternate swinging of the legs, while the third and the fourth kMPs are related to the vertical motion of the pelvis (generated by bending the knees). The combination of the effects of the single kMPs results in the complex human motion.

As highlighted in Section 3.5, an important possible application for this research is in the field of humanoid robotics (Ijspeert et al., 2002; Degallier et al., 2008; Ijspeert, 2008; Moro et al., 2011, 2012). The tests performed to use the kMPs to transfer the features of human gait to the walk of a robot were successful, but some further development is still required to achieve a full usability. At the current state valid joint trajectories were reconstructed from kMPs and applied on the COMAN robot (Tsagarakis et al., 2011), that could perform a human-like walking. The characteristics of the gait obtained, though, are fixed: to change the walking velocity, or other features, brand new trajectories need to be reconstructed. As an extension of this work it would be interesting to find a way to scale the joint trajectories to automatically change the features of the gait. This would involve future work on a study on the transitions from one gait to the other. At the current time, only steady-state gaits have been considered. An analysis on how the scaling coefficients of the kMPs [matrix S in equation (5)] change when the gait has a transition (e.g., from walking to running) could be useful to provide a better understanding of the correlation between kMPs and joint trajectories, and may lead to a complete application of the kMPs to generate a human-like robot walk.

This would reduce the gap between the motor performance of humans and robots, reproducing a criterion observed in the human behavior to control a complex system like a humanoid robot.

## Conflict of Interest Statement

The authors declare that the research was conducted in the absence of any commercial or financial relationships that could be construed as a potential conflict of interest.

## Appendix

The tables that follow are an extended version of those presented in Section 3: all the partial results of the comparative statistical analysis are reported.

**Table A1:** **Walking at low velocity kMPs (5 subjects)**.

	1st	2nd	3rd	4th	5th	Average	Sum	Weighted
Var Subj1_WLS	0.6775	0.1859	0.1084	0.0151	0.0069	/	0.9938	/
Var Subj2_WLS	0.5917	0.2037	0.1322	0.0478	0.0113	/	0.9869	/
Var Subj3_WLS	0.5921	0.2749	0.0993	0.0210	0.0086	/	0.9960	/
Var Subj4_WLS	0.5002	0.2517	0.1810	0.0354	0.0163	/	0.9846	/
Var Subj5_WLS	0.5206	0.2679	0.1519	0.0346	0.0131	/	0.9881	/
Var average	0.5764	0.2368	0.1346	0.0308	0.0112	/	0.9899	/
Xcov Subj1_Subj2	0.9945	0.9554	0.9583	0.9491	0.7377	0.9190	/	0.9751
Xcov Subj1_Subj3	0.9590	0.8811	0.9600	0.9111	0.8974	0.9217	/	0.9397
Xcov Subj1_Subj4	0.9827	0.9924	0.9795	0.9673	0.9235	0.9691	/	0.9833
Xcov Subj1_Subj5	0.9616	0.9280	0.9750	0.9488	0.9425	0.9512	/	0.9552
Xcov Subj2_Subj3	0.9532	0.8572	0.9883	0.8694	0.7705	0.8877	/	0.9292
Xcov Subj2_Subj4	0.9762	0.9632	0.9721	0.9543	0.7029	0.9137	/	0.9677
Xcov Subj2_Subj5	0.9555	0.8593	0.9651	0.9526	0.7996	0.9064	/	0.9318
Xcov Subj3_Subj4	0.9260	0.8795	0.9609	0.8846	0.8783	0.9058	/	0.9138
Xcov Subj3_Subj5	0.9866	0.9678	0.9640	0.8759	0.9453	0.9479	/	0.9750
Xcov Subj4_Subj5	0.9526	0.9306	0.9831	0.9793	0.9084	0.9508	/	0.9508
Xcov WLS	0.9648	0.9215	0.9706	0.9292	0.8506	0.9273	/	0.9527
Delay Subj1_Subj2	−0.0100	0	0.0200	0.0100	0.0500	0.0180	/	0.0096
Delay Subj1_Subj3	0.0500	0.0300	−0.0100	−0.0100	−0.0100	0.0220	/	0.0402
Delay Subj1_Subj4	0	0	0.0200	0	0	0.0040	/	0.0029
Delay Subj1_Subj5	0.0500	0.0200	0.0200	0	0	0.0180	/	0.0374
Delay Subj2_Subj3	0.0700	0.0300	−0.0300	−0.0200	−0.0800	0.0460	/	0.0540
Delay Subj2_Subj4	0	0	0	0	−0.0600	0.0120	/	0
Delay Subj2_Subj5	0.0600	0.0300	0	0	−0.0600	0.0300	/	0.0417
Delay Subj3_Subj4	−0.0400	−0.0200	0.0300	0.0200	0.0200	0.0260	/	0.0324
Delay Subj3_Subj5	0	0	0.0400	0.0200	0.0100	0.0140	/	0.0057
Delay Subj4_Subj5	0.0400	0.0200	0	0	−0.0100	0.0140	/	0.0261
Delay WLS	0.0320	0.0150	0.0170	0.0080	0.0300	0.0204	/	0.0249

**Table A2:** **Walking at low and high velocity kMPs (5 subjects)**.

	1st	2nd	3rd	4th	5th	Average	Sum	Weighted
Var WLS	0.5764	0.2368	0.1346	0.0308	0.0112	/	0.9899	/
Var WHS	0.5029	0.2811	0.1626	0.0311	0.0127	/	0.9902	/
Var average	0.5397	0.2590	0.1486	0.0310	0.0119	/	0.9900	/
Xcov WLS_WHS	0.9955	0.9826	0.9884	0.9520	0.9551	0.9747	/	0.9894
Delay WLS_WHS	−0.0200	0	−0.0200	0	0	0.0080	/	0.0139

**Table A3:** **Walking at low and high velocity and running kMPs (3 subjects)**.

	1st	2nd	3rd	4th	5th	Average	Sum	Weighted
Var WLS	0.6346	0.1948	0.1203	0.0315	0.0091	/	0.9903	/
Var WHS	0.4876	0.3092	0.1506	0.0346	0.0092	/	0.9911	/
Var RUN	0.4658	0.3049	0.1897	0.0176	0.0129	/	0.9909	/
Var average	0.5294	0.2697	0.1535	0.0279	0.0104	/	0.9908	/
Xcov WLS_WHS	0.9857	0.9872	0.9851	0.9108	0.8829	0.9503	/	0.9826
Xcov WLS_RUN	0.9848	0.9158	0.9489	0.7711	0.8761	0.8994	/	0.9553
Xcov WHS_RUN	0.9911	0.8884	0.9680	0.8817	0.7668	0.8992	/	0.9500
Xcov WLS_WHS_RUN	0.9872	0.9305	0.9673	0.8545	0.8419	0.9163	/	0.9635
Delay WLS_WHS	0	0	−0.0400	−0.0300	−0.0200	0.0180	/	0.0067
Delay WLS_RUN	−0.0600	0	−0.0800	−0.0700	0.0300	0.0480	/	0.0479
Delay WHS_RUN	−0.0700	−0.0200	−0.0400	−0.0200	0.0600	0.0420	/	0.0479
Delay WLS_WHS_RUN	0.0433	0.0066	0.0533	0.0400	0.0367	0.0360	/	0.0347

**Table A4:** **Walking with no constraint, holding an empty box and holding a 5 kg load kMPs (3 subjects)**.

	1st	2nd	3rd	4th	5th	Average	Sum	Weighted
Var WHS	0.4876	0.3092	0.1506	0.0346	0.0092	/	0.9911	/
Var WHSB	0.5332	0.2524	0.1621	0.0290	0.0113	/	0.9880	/
Var WHS5	0.5180	0.2476	0.1772	0.0372	0.0088	/	0.9889	/
Var average	0.5129	0.2697	0.1633	0.0336	0.0097	/	0.9893	/
Xcov WHS_WHSB	0.9907	0.9576	0.9801	0.9805	0.8910	0.9600	/	0.9783
Xcov WHS_WHS5	0.9885	0.8826	0.9113	0.9320	0.8373	0.9103	/	0.9425
Xcov WHSB_WHS5	0.9983	0.9704	0.9721	0.9646	0.9783	0.9767	/	0.9854
Xcov WHS_WHSB_WHS5	0.9925	0.9369	0.9545	0.9590	0.9022	0.9490	/	0.9566
Delay WHS_WHSB	0.0300	0	0.0100	0	−0.0100	0.0100	/	0.0172
Delay WHS_WHS5	0.0300	0	0.0100	0	−0.0100	0.0100	/	0.0170
Delay WHSB_WHS5	0	0	0	0	0		/	0
Delay WHS_WHSB_WHS5	0.0200	0	0.0067	0	0.0067	0.0067	/	0.0116

**Table A5:** **Reaching a target with the left hand (2 subjects)**.

	1st	2nd	Average	Sum	Weighted
Var Subj1_BallUp_Left	0.7498	0.2067	/	0.9565	/
Var Subj2_BallUp_Left	0.8904	0.0953	/	0.9857	/
Var average	0.8201	0.1510	/	0.9711	/
Xcov BallUp_Left	0.9662	0.9253	0.9457	/	0.9598
Delay BallUp_Left	0	0	0	/	0

**Table A6:** **Reaching a target with the left and the right hand (2 subjects)**.

	1st	2nd	Average	Sum	Weighted
Var BallUp_Left	0.8201	0.1510	/	0.9711	/
Var BallUp_Right	0.8451	0.1154	/	0.9605	/
Var average	0.8326	0.1332	/	0.9658	/
Xcov BallUp	0.9693	0.8745	0.9219	/	0.9562
Delay BallUp	0	0.0100	0.0050	/	0.0014

**Table A7:** **Reaching a target with the left hand while walking (2 subjects)**.

	1st	2nd	3rd	4th	Average	Sum	Weighted
Var Subj1_WLSBallUp_Left	0.7656	0.0913	0.0682	0.0277	/	0.9529	/
Var Subj2_WLSBallUp_Left	0.7883	0.0980	0.0758	0.0227	/	0.9848	/
Var average	0.7770	0.0946	0.0720	0.0277	/	0.9689	/
Xcov WLSBallUp_Left	0.9347	0.6070	0.8971	0.8697	0.8271	/	0.9004
Delay WLSBallUp_Left	−0.0200	−0.0500	−0.0700	−0.0100	0.0375	/	0.0264

**Table A8:** **Reaching a target with the left and the right hand while walking (2 subjects)**.

	1st	2nd	3rd	4th	Average	Sum	Weighted
Var WLSBallUp_Left	0.7770	0.0946	0.0720	0.0277	/	0.9689	/
Var WLSBallUp_Right	0.7903	0.0845	0.0633	0.0275	/	0.9656	/
Var average	0.7837	0.0895	0.0677	0.0276	/	0.9672	/
Xcov WLSBallUp	0.9827	0.9759	0.9862	0.8654	0.9526	/	0.9803
Delay WLSBallUp	0	0	0	−0.0200	0.0050	/	0.0006

**Table A9:** **Combination of the kMPs of walking and the kMPs of reaching (2 subjects)**.

	1st	2nd	3rd	4th	Average	Sum	Weighted
WLS	/	/	1st kMP	4rd kMP	/	/	/
BallUp	1st kMP	2nd kMP	/	/	/	/	/
Var WLSBallUp	0.7837	0.0895	0.0677	0.0276	/	0.9672	/
Xcov Combination	0.9762	0.8085	0.9480	0.9339	0.9167	/	0.9588
Delay Combination	0	0.0300	0.0700	0	0.0250	/	0.0077

### Highlights

- Data for periodic motions (locomotion) and discrete motions (reaching for a target with one hand) of human subjects, as well as motions that are a combination of discrete and periodic movements (reaching for a target while walking), are collected with a motion capture system.
- A Principal Component Analysis (PCA) is applied on the joint trajectories derived from the data of the subjects, and a comparative analysis on the components extracted is performed.
- It is shown that the first five components extracted from the periodic motions (99% of cumulative variance explained) are invariant to the subjects, to the walking/running velocity, and to the constraints on motion of the arms imposed by asking the subjects to hold an object with both hands.
- In the same way, the first two components extracted from the discrete motions (95% of cumulative variance explained) are invariant to the subjects, and to the hand used to reach for the target.
- These two invariant sets of signals identified are named kinematic Motion Primitives (kMPs).
- A further analysis shows that the kMPs extracted from periodic motions and those extracted from discrete motions can combine to describe those movements that are a combination of simultaneous periodic and discrete motions.
- As a possible application to this theoretical study of human motion, it is shown that the kMPs can be used to generate human-like trajectories for a humanoid robot.

## Abbreviations

COMAN, COmpliant huMANoid robot under development at IIT; CPG, central pattern generators; kMPs, kinematic motion primitives; MPs, motor primitives; PCA, principal component analysis.
